# Acceptability of Integrated Vector Management of *Aedes* in the iDEM Project: Findings from a Community-Based Survey in Urban Malaysia

**DOI:** 10.3390/tropicalmed11070199

**Published:** 2026-07-15

**Authors:** Neal Alexander, Nurulhusna Ab Hamid, Farah Diana Ariffin, Muriel Rabilloud, Tim W. R. Möhlmann, Frédéric Schmitt, Jason H. Richardson, Mitra Saadatian-Elahi

**Affiliations:** 1 International Statistics and Epidemiology Group, Department of Infectious Disease Epidemiology and International Health, London School of Hygiene and Tropical Medicine, Keppel Street, London WC1E 7HT, UK; 2 Medical Entomology Unit, Institute for Medical Research, National Institutes of Health, Ministry of Health, Setia Alam 40170, Selangor, Malaysia; husna.hamid@moh.gov.my (N.A.H.); farahdiana.a@moh.gov.my (F.D.A.); 3 Laboratoire de Biométrie et Biologie Évolutive, Équipe Biostatistique-Santé, CNRS, UMR 5558, F-69100 Villeurbanne, France; muriel.rabilloud@chu-lyon.fr; 4 Service de Biostatistique et Bioinformatique, Pôle Santé Publique, Hospices Civils de Lyon, Université de Lyon, F-69000 Lyon, France; 5 In2Care B.V., Marijkeweg 22, 6709 PG Wageningen, The Netherlands; tim.mohlmann@envu.com; 6 Envu, 1 Place Giovanni Da Verrazzano, F-69009 Lyon, France; frederic.schmitt@envu.com; 7 Innovative Vector Control Consortium, Pembroke Place, Liverpool L3 5QA, UK; jason.richardson@ivcc.com; 8 Centre International de Recherche en Infectiologie (CIRI), Inserm U1111, CNRS UMR5308, ENS de Lyon, Lyon 1 University, CEDEX 07, F-69364 Lyon, France; 9 Service Hygiène, Epidémiologie et Prévention, Centre Hospitalier Hôpital Edouard Herriot, Hospices Civils de Lyon, F-69437 Lyon cedex, France

**Keywords:** dengue, integrated vector management, residual spraying, autodissemination devices, cluster randomized trial, barriers, facilitators

## Abstract

For dengue, there is little evidence regarding community-level acceptance, facilitators and barriers for integrated vector management (IVM). We carried out a survey to measure these aspects in the context of a large cluster-randomized controlled trial of IVM against dengue vectors in urban Malaysia, in which the component control methods were (i) outdoor residual spraying, (ii) autodissemination devices (ADDs) and (iii) community engagement. The survey population comprised (i) members of the Joint Management Body (JMB) of building developments in both arms, and (ii) residents in the intervention arm. Members of both groups were requested to complete a structured questionnaire, through face-to-face interviews. Among JMB responders, 138 were the intervention clusters, and 94 from those receiving routine activities (control arm), and there were 604 responders among residents of intervention clusters. Among the JMB members, safety of the control methods (spraying and ADDs) was among the most important potential facilitators. Among barriers, the ones identified by over one-third of the participants were lack of information about the iDEM IVM methods, lack of communication and of involvement with the iDEM project personnel. Overall, the iDEM IVM methods were seen positively, and investment by the trial in community engagement may have contributed to this.

## 1. Introduction

Vector-borne arboviral diseases such as dengue, West Nile viruses and chikungunya have a huge burden of morbidity and mortality worldwide [1]. In the absence of effective vaccines, vector control is the main tool to fight against these infectious diseases [2]. However, the challenging threat of insecticide-resistant vectors, and global climate/environmental changes constitutes a veritable barrier in the prevention and management of these life-threatening diseases [3,4].

The WHO defines integrated vector management IVM as a: “rational decision-making process for the optimal use of resources for vector control. IVM instead of a single vector control tool has been advocated by the World Health Organisation (WHO). For sustainable impact, vector control needs to cross and integrate multiple sectors and disciplines linking efforts in environmental management, health education, governance and vector control. IVM relies on a smart combination of vector control methods adapted to the local epidemiology of vector-borne diseases.

Despite the WHO recommendation on the use of IVM for more than two decades, the deployment of this concept in countries endemic for vector-borne diseases remains low. Lack of innovative tools, insufficient political advocacy and limited human capacity are listed as the key barriers of the use of IVM [5]. In October 2016, the Malaysian Ministry of Health, WHO Western Pacific Regional Office and its Collaborating Centres organized a training on the use of IVM as an intervention strategy for the control of dengue and other arboviral diseases [6]. The Malaysian Ministry of Health routinely practices IVM using combinations of source reduction, larviciding, space spraying, environmental management and education as a reactive strategy. The intervention for dengue epidemiology in Malaysia (iDEM project), was initiated in 2020 to complement and enhance this approach by looking at a more proactive, preventive IVM program [7,8]. The primary objective of this cluster randomized controlled trial was to assess the effectiveness of the proposed IVM on dengue incidence in urban Malaysia [7]. Overall, 139 and 141 clusters were randomly allocated to the control (routine activities) or the intervention arm in a 1:1 ratio. The iDEM intervention program included both chemicals and biologicals, i.e., targeted outdoor residual spraying using K-Othrine^®^ Polyzone (Envu, Lyon, France) and deployment of auto-dissemination devices (ADDs, In2Care^®^, Wageningen, Netherlands), with different modes of actions targeting various mosquito life-cycle stages in conjunction with community engagement [7]. Clusters in the intervention arm received the proactive IVM in addition to the routine Ministry of Health vector control activities, while clusters in the other arm received only the routine vector control activities [7].

A better understanding of the population’s feedback about various topics related to a vector control project and their level of satisfaction with the intervention program would enable the healthcare professionals to learn and to improve such interventions and can inform policymakers on how to widen the program. We carried out a survey among inhabitants of the iDEM intervention clusters to evaluate knowledge and attitudes towards the iDEM program and its related interventions.

The main objective of this population-based survey was to evaluate perceived barriers, facilitators and attitudes towards the iDEM program and its related vector control methods. The secondary objectives consisted of collecting information about (i) the trusted source of information, (ii) participation of the respondents in vector control activities and (iii) the level of nuisance provoked by mosquitoes in their property.

## 2. Materials and Methods

### 2.1. Study Areas and Survey Collection Strategy

The survey was conducted in iDEM intervention clusters in the Federal Territory of Kuala Lumpur and Putrajaya, Malaysia, from April 2022 until December 2022. The iDEM clusters consist of medium- and low-cost high-rise buildings. The study population was composed of two groups. The first was local residents of the iDEM intervention localities, living there February 2020–September 2022 (*n* = 604). This sample size gives a binomial standard error of 2% if the proportion selecting any question option is 50%, with this standard error being lower for any other proportion. The second group was members of the Joint Management Body (JMB) in localities allocated either to intervention or to routine activities (*n* = 138 and *n* = 94 respectively). In Malaysia, JMB is a group formed to manage the common property of an area that has multiple owners. Vector control management is part of the JMB duties in each locality.

The target sample size in each locality was achieved by opportunity sampling, with study personnel requesting participation from residents who were visible in common areas. They were requested to answer survey questions which were administered using a structured questionnaire, through face-to-face interviews. The numbers of non-responders were not recorded, nor were their characteristics, so it is not possible to compare responders and non-responders. All the available questionnaires were complete, with no missing data.

JMB respondents were sought from those holding the following roles: JMB chairperson, JMB committee member, manager, technician and management executive. They were requested to answer survey questions which were administered using a structured questionnaire, through face-to-face interviews.

All interviewers were trained for one week before the interview session started to ensure that the objectives, methodology, expectations and questions were well understood. A pretest including 30 persons was conducted in both medium- and low-cost localities to improve the usability and comprehension of final version.

The closed-ended questionnaire consists of a structured questionnaire which was administered through face-to-face interviews and was composed of several parts including socio-demographic data (age, gender, ethnicity, education level, occupation and housing type), knowledge of the iDEM project and general information about the trusted source of information, the way they participate in vector control activities and the level of nuisance provoked by mosquito in their property. JMBs had to answer a series of additional questions.

To measure knowledge related to the methods used during the iDEM trial, i.e., community engagement, outdoor residual spraying and deployment of autodissemination devices, participants could choose between three response categories: “yes,” “no,” and “don’t know.” Feedback on different iDEM vector control methods was measured by 29 items on a five-point Likert scale that ranged from 1 (strongly disagree) to 5 (strongly agree). The overall trial was rated by 6 questions using a five-point scale ranging from one (very poor) to five (excellent). Self-reported practice towards *Aedes* mosquito preventive measures, the trusted sources of information and the level of mosquito nuisance were assessed using 12 items each.

The JMB section included open questions about positive feedback or complaints they had received from the residents, the use of additional private vector control activities in the past two years and what should be improved to increase the population to adhere to the iDEM vector control methods. Close-ended questions consisted of information about barriers for the population to adhere to the vector control methods, the most frequent method that was used to transmit the iDEM communication materials to the population and how they would rate the overall iDEM trial.

JMB personnel were included from both arms because they had a clearer overall concept of the trial objectives, even when not receiving the control methods. By contrast, we wanted to ask residents about concrete experience with the iDEM vector control methods, which was not possible in the localities which received only governmental routine activities.

### 2.2. Statistical Analysis

The presentation of the results is largely descriptive. In addition, responses were compared between low- and medium-cost areas, these being strata in the randomised trial. When the responses were chosen from five ordered options, the results were compared between strata (low- and medium-cost) using the Mann–Whitney test. To allow for multiple testing, the Holm procedure was used [9], with an overall two-sided significance level of 5%, and the multiplicity defined for each set of questions, e.g., 15 for the perceived facilitators of IDEM methods according to JMB. The Spearman (non-parametric) correlation coefficient was used to examine associations between variables. Analysis was done in R version 4.4.2 (R Foundation for Statistical Computing, Vienna, Austria).

Generative artificial intelligence (AI) was not used to generate manuscript text, data, figures or graphics, nor to assist in study design, data collection, statistical analysis planning or interpretation of results. AI was used to support the coding of the planned statistical analyses.

## 3. Results

**Figure 1:**
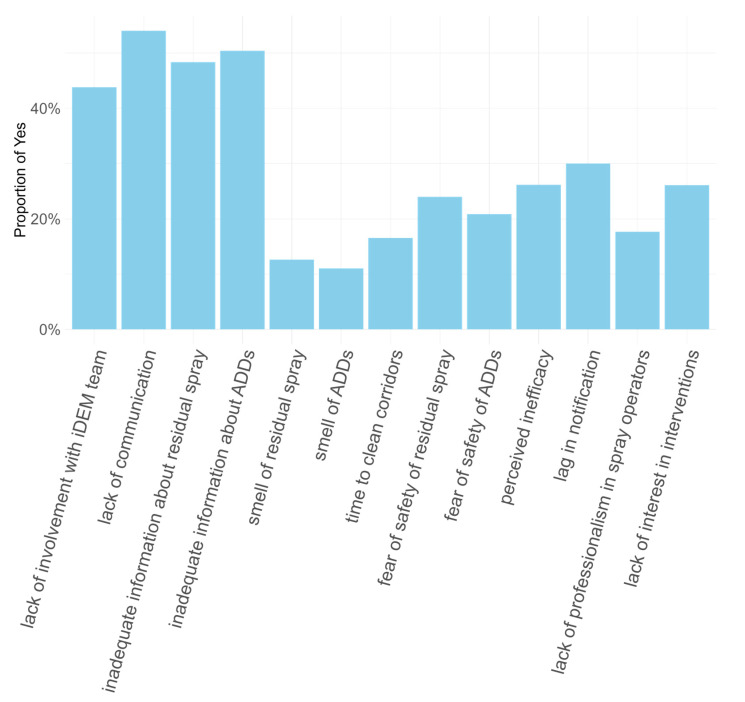
Perceived barriers to IDEM intervention according to JMB.

Table 1 shows demographic and other characteristics of the two populations of the study: building managers (JMB) and residents. Both groups had an even male:female split, were majority Malay and had a median age group of 40–60 years. Figure 1 shows barriers to implementation of the iDEM vector control methods, as perceived by JMB members. Of the 13 options, the ones which were identified by over one-third of the participants were a lack of information about the vector control methods (outdoor residual spray and ADDs), lack of communication and of involvement with the iDEM project personnel. Characteristics and efficacy of the vector control methods were among the potential concerns identified by less than a third of participants. These four variables were also identified by more than a third of participants in each cost stratum. The only additional such variable was a lag in the notification of results in the low-cost stratum.

**Figure 2:**
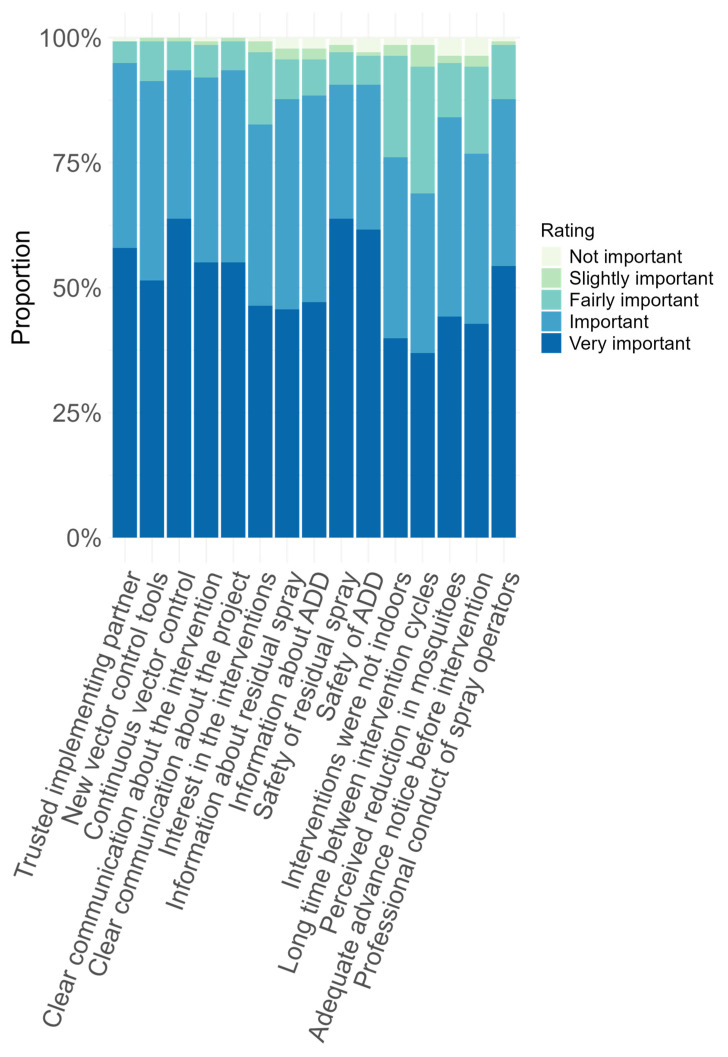
Perceived facilitators of IDEM vector control methods according to JMB.

Conversely, safety of the component of vector control methods was among the most important potential facilitators, in terms of the proportion of JMB members rating it as “Very important” (Figure 2). All 15 potential facilitators had a median score of “Important” or “Very important”, overall and within each stratum. The ones with the highest proportion of the less favourable scores (from “Fairly important” to “Not important”) were i) the location of the vector control methods outside residences (in common areas) and ii) the long time between intervention cycles. The percentage of JMB members that would “Definitely” or “Very probably” continue to use the iDEM vector control methods was 76.1% (105/138) for outdoor residual spray and for ADDs, and 66.7% (92/138) for community engagement. There was no statistically significant difference between strata (low- and medium-cost) for any component. More generally, comparing the five-level scores of the 15 potential facilitators between strata, there were no statistically significant differences.

**Figure 3:**
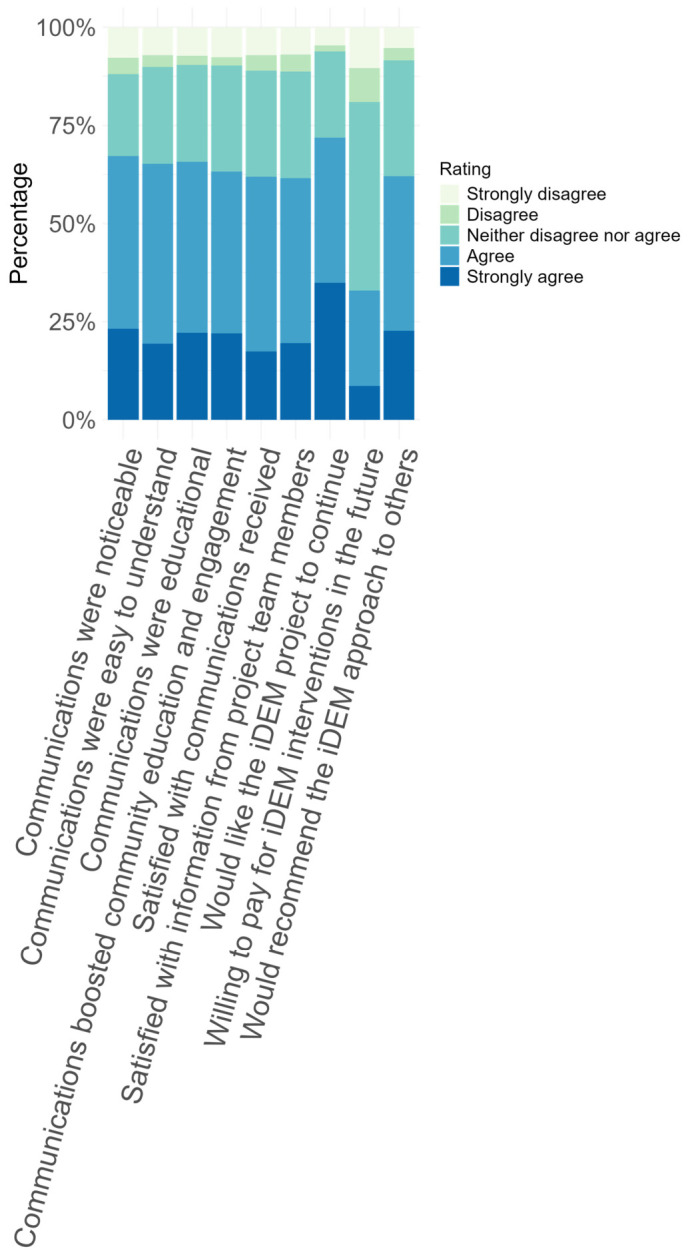
Perceived community engagement facilitators of the IDEM intervention according to residents.

Figure 3 shows the success, as perceived by residents, of the intended community engagement facilitators of the intervention, as well as attitudes to possible future actions Answers to the engagement questions were similar, with all having the median response of “Agree”. In summary, residents agreed that the communication materials were visible, easy to understand and educational. In terms of future actions, the median response was again “Agree” in terms of wanting the intervention to continue and recommending them to others. However, there was less willingness to pay for the iDEM vector control methods in the future, with the median score being “Neither disagree nor agree”. For these questions, agreement tended to be stronger in the low-cost areas than in the medium-cost ones, and this was statistically significant for eight of the nine questions (allowing for multiple comparisons with the Holm procedure).

**Figure 4:**
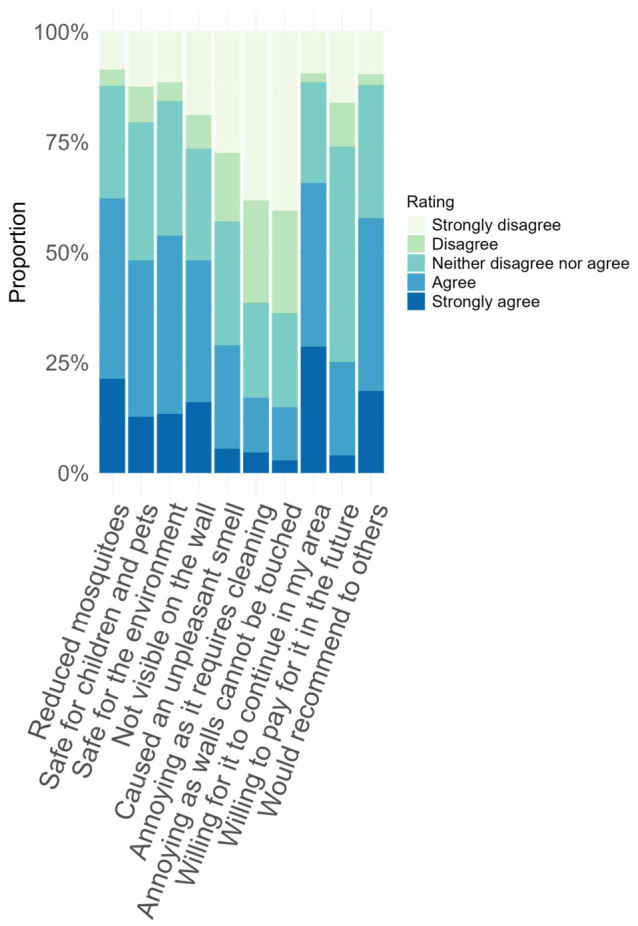
Residents’ beliefs and attitudes regarding outdoor residual spraying.

Figure 4 shows residents’ beliefs and attitudes to residual spraying. Three groups of questions can be distinguished. Bars 1–4 are positive beliefs, 5–7 are negative beliefs, and the final three are attitudes to possible future actions. In terms of the four questions reflecting positive beliefs, in particular safety, the median responses were “Agree” (2/4) or “Neither disagree nor agree” (2/4). For the three negative beliefs, in particular inconvenience, the median responses were “Disagree” (2/4) or “Neither disagree nor agree” (1/4). In terms of correlations within and between the three groups of variables, the average Spearman correlation within each group ranged from 0.51 to 0.57. For the three cross-group comparisons, the average correlations were lower, ranging from 0.08 to 0.44. Residents in the low-cost stratum were statistically significantly more willing for residual spraying to continue in their area. Allowing for the multiple comparisons, this was the only variable to show a statistically significant difference.

**Figure 5:**
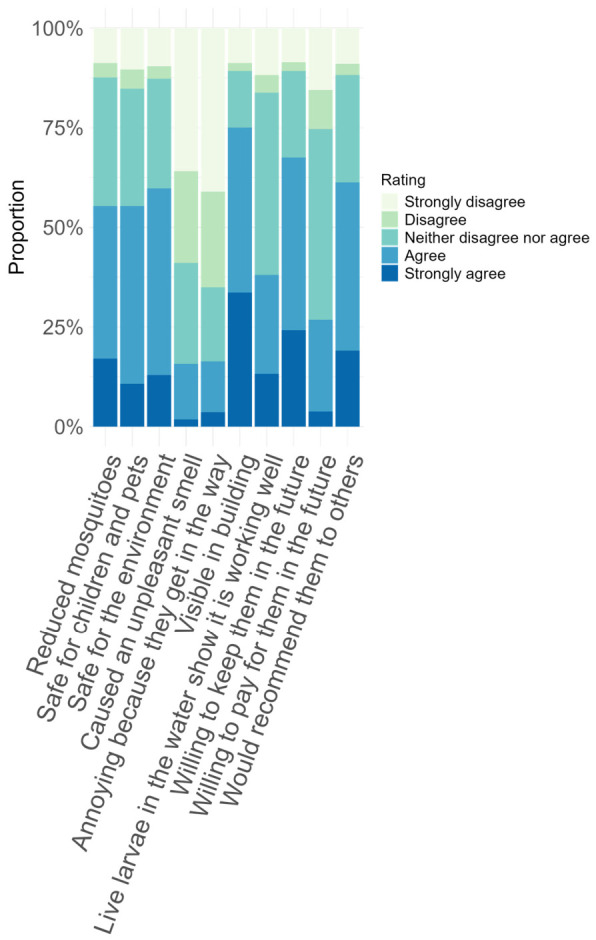
Residents’ beliefs and attitudes regarding the autodissemination devices.

Similarly, Figure 5 shows residents’ beliefs and attitudes to ADDs. Five groups of questions can be distinguished: bars 1–3 are positive beliefs, 4 and 5 are negative beliefs, 6 and 7 refer to visibility and under-standing of the device function and the final three are attitudes to possible future actions. All three questions reflecting positive beliefs had “Agree” as their median responses, and for both negative beliefs, it was “Neither disagree nor agree” (1/3). In terms of correlations within and between the five groups of variables, the average Spearman correlation within the three positive beliefs was 0.67, with the next highest average being 0.52 (between the first and third groups), and most of the other averages were between 0.46 and 0.52. Overall, the responses themselves are similar to those about outdoor residual spraying. In terms of future actions, the median responses for both spraying and ADDs were the same as in Figure 3. Comparing the strata, the only statistically significant difference was that people in the low-cost stratum agreed more strongly with having seen the devices in their buildings.

The present survey showed that the proactive iDEM strategy was acceptable to both building managers (JMB) and residents. In addition, these groups expressed their willingness to continue the intervention.

## 4. Discussion

Public comprehension and acceptability of preventive programs determine community support and facilitate policy implementation.

In Malaysia, JMBs maintain and manage subdivided buildings and common property. Therefore, they could play an active and influential role on the conduct and acceptability of a preventive vector control program. The observed acceptability of the program by the residents could be explained, at least partly, by the fact that JMBs were the first contact point during the overall iDEM intervention period [7,10]. On the other hand, the acceptability by JMBs could be due to trust and respect for the government-related entity (Institute for Medical Research) that ran the program.

Perceptions of risks posed by insecticides to human and animal health could reduce the adherence of the population to preventive vector control campaigns. The safety of the iDEM vector control tools [11,12] was among the major facilitators of the intervention.

A systematic review of the effectiveness of indoor spraying as a dengue vector control method [13] found that “Few studies included in the review reported qualitative estimates of community acceptance”. Spraying, as we did, in semi-indoor areas avoids some of the commonly reported barriers to indoor residual spraying, in particular the need to move furniture outside the residence [14,15,16]. For indoor spraying, negative perceptions of the behaviour and communication of spraying operatives may severely reduce the acceptability of indoor spraying [15,17,18], so the positive perceptions of such personnel were a strength of the current trial.

The few existing studies on the acceptability of autodissemination devices emphasize the importance of community-level acceptance [19,20]. A more general review of pyriproxyfen [21] found that “Community participation and acceptance has not consistently been successful and needs to be further assessed.” A study in Texas [22] found that acceptance is not necessarily inherent to individuals or even single families but rather may be determined at a wider level. In the current trial, we tried to ensure community-level acceptance via engagement with building managers, and educational materials target more widely, and, whether or not because of these measures, the acceptability of the ADDs was generally high.

Clear, comprehensive and regular communication to the population is also a cornerstone of a successful acceptance of a vector control program. During the first weeks of the start of our trial, i.e., before the occurrence of the COVID-19 pandemic [8], face-to-face meetings with JMBs and the residents were organised to inform the population about the trial. Due to the deployment of COVID-19 related lockdown, meetings with residents were not possible for some time, with the last Movement Control Order ending in May 2021 [10]. When these orders were in effect, communication was maintained through email, social media and WhatsApp, as well as letters informing residents about upcoming intervention activities.

One possible limitation of the current study is that the interviewers may not have been seen as independent of the trial, nor of public health authorities, and that this may have affected respondents’ answers. Moreover, the sampling method was not random, and we lack data on the proportion of non-response. Also, although carried out in the framework of a randomized trial, we have described absolute scores rather than between-arm comparisons. Indeed, randomized comparisons of residents’ attitudes to the control methods were not possible because only those in the intervention arm had knowledge of these methods, and residents in the other arm were not interviewed. In terms of generalizability, the two vector control methods may have roles in integrated management of dengue vectors in multi-occupancy buildings in urban areas, as long as there is effective communication with building residents and management. On the other hand, the generalizability may be affected by time between publication and the work being carried out, the latter being 2022. In this year, dengue incidence in Malaysia was the third lowest among the 10 years from 2016 to 2025 [23]. Strengths include the coverage of two types of stakeholders (building management and residents) and a high sample size.

The trial was stratified on whether the locality (housing development) was low- or medium-cost. In general, we did not find strong differences between these strata. However, it was notable that residents in low-cost localities were more positive about potential community engagement facilitators, expressed greater desire for the iDEM vector control methods to continue and showed greater willingness to pay for them in the future (Figure 3). Apartments in low-cost localities tend to be older, and with lower maintenance fees that make it less feasible to hire pest control companies, leaving the localities more reliant on government initiatives.

## 5. Conclusions

This is one of the few evaluations of community acceptance of dengue vector management. The study was part of an intervention trial, and overall, the deployed vector control methods were seen positively, by both building managers and residents, and in terms of both safety and convenience. Investment by the trial in community engagement may have contributed to this.

## Figures and Tables

**Table 1:** Summary statistics by respondent type and arm.

	Resident (Routine Activities Arm)	JMB (Routine Activities Arm)	JMB (Intervention Arm)
Median age group (years)	40–60	40–60	40–60
Male, number (%)	303 (50.2%)	45 (47.9%)	71 (51.4%)
Ethnicity			
Malay	501 (82.9%)	67 (71.3%)	98 (71%)
Chinese	29 (4.8%)	12 (12.8%)	20 (14.5%)
Indian	54 (8.9%)	15 (16%)	20 (14.5%)
Other	20 (3.3%)	0 (0%)	0 (0%)
JMB status: Committee member	-	21 (22.3%)	75 (54.3%)
JMB status: Manager	-	24 (25.5%)	35 (25.4%)
JMB status: Non-resident staff	-	49 (52.1%)	28 (20.3%)

## Data Availability

The database used for analysis will be available from the authors upon reasonable request and with permission of the Malaysian Ministry of Health.

## Abbreviations

The following abbreviations are used in this manuscript:

ADD	autodissemination device
iDEM	intervention for dengue epidemiology in Malaysia
IVM	integrated vector management
JMB	Joint Management Body
WHO	World Health Organisation
